# Continued influence of misinformation in times of COVID‐19

**DOI:** 10.1002/ijop.12805

**Published:** 2021-08-26

**Authors:** Dian van Huijstee, Ivar Vermeulen, Peter Kerkhof, Ellen Droog

**Affiliations:** ^1^ Department of Communication Science Vrije Universiteit Amsterdam Amsterdam The Netherlands

**Keywords:** COVID‐19 misinformation, Fact‐checking, Negativity bias, Continued influence effect, Persuasive impact

## Abstract

Health‐related misinformation, especially in times of a global health crisis, can have severe negative consequences on public health. In the current studies, we investigated the persuasive impact of COVID‐19‐related misinformation, and whether the valence of the misinformation and recipients' degree of overconfidence affect this impact. In two pre‐registered experimental studies, participants (*N* = 403; *N* = 437) were exposed to either a positive or a negative news article describing a fictional hospital's high COVID‐19 recovery/mortality rates. Half of the participants subsequently received a correction. Attitudes towards the hospital were measured before and after exposure. Results of both studies showed that, as expected, corrections reduced the persuasive impact of misinformation. But whereas some persuasive impact remained for corrected negative misinformation (a continued influence effect), it reversed for corrected positive information, causing people to have more negative attitudes towards the hospital than before exposure to any information (a backfire effect). These results corroborate prior suggestions that continued influence effects are asymmetric: negative misinformation is harder to neutralise than positive misinformation. Participants' overconfidence degrees did not have a moderating role in misinformation effects. Even though corrections decrease the persuasive impact of health‐related misinformation, continued influence remains for negative misinformation.

Even in times when receiving correct information matters most, such as during the COVID‐19 pandemic, we may still be exposed to information that is untrue (e.g., “Chinese created COVID‐19 as a biological weapon” or “COVID‐19 no more deadly than seasonal flu”). According to the World Health Organization, COVID‐19 “has been accompanied by a massive *infodemic*.” This infodemic refers to an overabundance of (often inaccurate) information about COVID‐19 that creates difficulties for the public to differentiate facts from fiction (Erku et al., 2020, p. 2).

Believing and acting in accordance to misinformation can be harmful to public health. Several organisations fight the spread of misinformation by informing the public, providing tools to determine the reliability of messages, and fact‐checking. Numerous fact‐checking pages specialising in correcting COVID‐19 misinformation have been initiated by organisations, such as Johns Hopkins University and Europol. This raises the question: how effective are these corrections?

Correcting misinformation can be challenging. Several studies show that being consciously aware that information is corrected (and thus is misinformation), does not always neutralise its influence (Lewandowsky et al., 2012). This phenomenon is called the *continued influence* effect of misinformation, indicating that there seems to be a discrepancy between factual belief in misinformation (i.e., knowing and accepting that the information is incorrect) and the persuasive effects it still has on our behaviour and reasoning.

Interestingly, the effect of issued corrections seems to differ depending on the valence of the misinformation (Guillory & Geraci, 2016), causing an asymmetry in continued influence: Whereas corrections of positive information may reverse initial persuasive effects (Cobb et al., 2013; Nyhan & Reifler, 2010—however, see Wood & Porter, 2019 for an only partly successful replication), persuasive effects of negative information seem more resistant to corrections (e.g., Ecker et al., 2019; Gordon et al., 2017). Hence, the valence of misinformation can determine its persistence and probable consequences. Understanding these (potentially asymmetrical) effects of misinformation and raising awareness about their consequences is important in the fight against misinformation—especially in health crises such as COVID‐19, where misinformation is prevalent and may have particularly harmful effects.

Theoretically, our study seeks to provide further evidence that continued influence effects of positive and negative misinformation may be asymmetrical. Additionally, we investigate overconfidence as an individual factor that might explain continued influence effects.

## Continued influence of misinformation and the role of valence

The continued influence of misinformation effect, or in short “continued influence,” describes the notion that discredited information can continue to influence reasoning and understanding of recipients (Johnson & Seifert, 1994). This means that despite conscious awareness that certain information is not true, it might still affect recipients' rationale and actions. Consequently, correcting misinformation (e.g., through fact‐checking) may not completely neutralise its persuasive effects (e.g., Lewandowsky et al., 2012).

One factor that can affect the degree of continued influence of misinformation is its valence, that is, the degree to which the information portrays its object favourably or unfavourably (e.g., “Fauci's warnings saved millions” vs. “Fauci responsible for failing COVID crisis response”). Research by Cobb et al. (2013) shows that correcting positive (political) misinformation can be effective, resulting in the absence of continued influence, and that a correction can even reverse the initial positive persuasive effects. In contrast, research focusing on negative political misinformation did find continued influence after correction (Thorson, 2016), as did studies in other contexts (e.g., Ecker et al., 2019; Gordon et al., 2017). These findings suggest that the effectiveness of corrections may depend on the valence of the misinformation. So far, only a few studies have compared the effects of correcting positive versus negative information, most notably a study by Guillory and Geraci (2016), which, in a political context, finds that after correction, the reliance on positive or neutral misinformation is reduced almost completely, whereas the reliance on negative misinformation is reduced by about half.

A potential reason for this asymmetry can be that negative information is perceived as more attention‐grabbing and informative than positive information (i.e., the negativity bias; Baumeister et al., 2001). From an evolutionary perspective, this makes sense: Negative information can imply severe and irreversible risks (e.g., a plant's poisonousness). Attending to such information promotes survival, possibly more than positive information (e.g., a plant's nutritiousness; Vaish et al., 2008). Hence, the initial attention and information value attributed to negative information may outweigh that of positive information, making its impact harder to correct at a later stage.

## Individual factors influencing the effect of misinformation

One reason why we are inclined to believe misinformation is that in order to comprehend new information, we first accept the information as true (Gilbert et al., 1990; Lewandowsky et al., 2012). This process of temporary “believing” misinformation happens at a basic, unconscious, and intuitive level (Kahneman, 2011). Relative to the first stage in which information is accepted as true, unbelieving is a process that is characterised more by analytic thinking (Pennycook & Rand, 2020), an active process that takes time and conscious effort (Kahneman, 2011). This suggests that the effectiveness of corrections of misinformation may depend on recipients' motivations or opportunities to engage in effortful cognitive processing.

Prior knowledge and prior attitudes also play a role in the process of (un)believing misinformation. Mental models for new information that is in line with prior perceptions are likely easier to build; in contrast, a correction that is not in line with such perceptions will be more effortful to process and thus may lack effectiveness (Nyhan & Reifler, 2010).

Interestingly, recipients who have limited knowledge of a particular subject tend to overestimate their knowledge or abilities, for example as compared to established authorities such as doctors or scientists. This phenomenon is known as the Dunning‐Kruger effect (Dunning, 2011) or overconfidence (Pallier et al., 2010). Motta et al. (2018) showed that the less people knew about autism, the more likely they were to believe their knowledge exceeded that of medical professionals. Overconfidence also relates to judging misinformation as accurate (Pennycook & Rand, 2020). This may be, firstly, because overconfident people tend to be intuitive rather than reflective thinkers (even though they may rate themselves as being reflective), and secondly because they may be open to an impulse to assess information as true (Pennycook & Rand, 2020). We reason that this may also apply to COVID‐19 knowledge: overconfident recipients will be more susceptible to misinformation; hence, the correction of misinformation will be less effective, leading to higher degrees of continued influence. Since overconfidence tends to relate to anti‐establishment worldviews in news consumers (Van Prooijen & Krouwel, 2020), we expect the negative (critical) misinformation to be particularly effective, and its (“pro‐establishment”) correction to be particularly ineffective in overconfident participants. Hence, we expect overconfidence to boost the negativity bias of continued influence.

## The present studies

We aimed to investigate the persuasive impact of (corrected) COVID‐19‐related misinformation, and the moderating effects of misinformation valence and overconfidence. Because of our premise that the continued influence effect of misinformation may occur despite corrections, we were ethically restricted to use—as stimuli—COVID‐19‐related misinformation that is relatively benign and cannot put participants' health at risk. To that end, we exposed participants' to either a positive or negative news article concerning COVID‐19 recovery/mortality rates at a foreign hospital, followed by either a correction or no correction. The following pre‐registered hypotheses were tested: (a) COVID‐19‐related misinformation continues to have persuasive impact after correction; (b) this continued influence of misinformation is stronger for negative than for positive misinformation; (c) continued influence of misinformation is stronger for more overconfident news consumers; and (d) the negativity bias of continued influence is stronger for more overconfident news consumers.

## STUDY 1

A 2 (valence: positive vs. negative information) × 2 (no correction vs. correction) between‐subjects design was used to investigate the continued influence of COVID‐19‐related misinformation. A pre‐registration document can be found on the OSF (https://osf.io/jsu24/?view_only=8028d4a1194e4787a9187d4f49de8f33). The study was also checked for compliance to the home institute's ethical guidelines.

### Participants

Sample size was determined a priori using G*Power 3.1 based on H2. To test H2 with sufficient power (0.80), based on an estimated small‐to‐medium effect size of *d* = 0.40 for a *t*‐test (or, equivalent, *f* = 0.2 for an ANOVA), we required a sample of 100 respondents for the two groups that received a correction to the misinformation. We set the two groups that did not receive a correction to the same size, resulting in a total required sample of 400 respondents for four groups.^1^

Four Dutch BSc students recruited a convenience sample of Dutch‐speaking participants through social media (WhatsApp, Facebook, Instagram). Responses were collected between 27 May and 17 June 2020—a time characterised in The Netherlands by a relaxation of COVID‐measures. To illustrate, on 1 June, access to COVID‐tests became available to everyone, and on 3 June, primary schools reopened. Thirty‐two participants with a total response time under 5 and over 40 minutes (too much time between stimulus and response) were excluded from the analyses. Also, participants who did not meet the age limit (≥18 years old) and/or who did not complete the survey (*n* = 181) were removed. All other participants were included, resulting in a sample of 403 participants with an average age of 35.6 years (range 18–80, *SD* = 16.4), 67.7% female. Education was relatively high: 34.7% had a university degree.

### Stimuli

The stimuli used in this experiment consisted of a news article about a hospital and a correction (for details, see Supporting Information Study 1).

#### News article

Two different news articles about a fictional German hospital, Städtisches Klinikum Düsseldorf, were created. Both were supposedly from a (fictional) Dutch news source. The positive article (*n* = 202) included a story about the hospital winning an award for having the highest COVID‐19 recovery rates in Western Europe. It was additionally praised for accommodating Dutch COVID‐19 patients. The negative news article (*n* = 201) reported on the hospital having the highest COVID‐19 mortality rates of Western Europe, and a lack of available ICU beds. Both articles had a similar layout.

#### Correction

For both the positive and negative article, a correcting message was created. In both conditions, it stated that authorities declared the information in the article about recovery/mortality rates and available ICU beds to be incorrect. Both corrections had a similar layout and a large title showing “Correction” (205 participants received a correction; 198 did not).

### Procedure

After providing informed consent and answering demographic questions, participants provided their impression (general impression, and expected quality of care) of five fictional hospitals based on their name and an image of the hospital building. One hospital was presented per page, and the order of presentation was randomised. Subsequently, participants were randomly assigned to a positive or negative news article about one of these hospitals, the Städtisches Klinikum Düsseldorf. To prevent participants from skipping the article, the “next” button only appeared after 15 seconds. Subsequently, participants indicated perceived valence of the article (a slider ranging from *very negative* [−5] to *very positive* [5]), and answered seven state‐anxiety questions and four questions measuring COVID‐19 concern (both state‐anxiety and COVID‐19 concern were included for exploratory reasons^2^). For half of the participants, a correction followed in which the previous article was debunked; the other half received no correction. Subsequently, participants indicated to what extent they thought the information provided in the original article was true (a slider ranging from *certainly not true* [−5] to *certainly true* [5]). Then, a second evaluation task followed, in which participants re‐evaluated the same five hospitals, again in random order. Only evaluations of the focal hospital were used; the other measurements served as distractors to obscure the relationship between the stimulus materials and the dependent measure. Participants then rated hospital reputation (also included for exploratory reasons), followed by 25 items assessing COVID‐19 overconfidence. Finally, participants were debriefed—here, it was emphasised that all stimuli were entirely fictional—and thanked for their time.

### Measures

#### Persuasive impact

To measure the misinformation's persuasive impact, all participants evaluated the focal hospital before (T1) and after (T2) exposure to the stimuli, and these scores were compared. The evaluation consisted of two parts: general impression of the hospital, and expected quality of care. Both were measured using a slider ranging from very negative (−5) to very positive (5).

Persuasive impact was computed as the absolute difference between the evaluation at T1 and T2, |T2 − T1|. However, if the difference was not in the direction intended by the news article (e.g., if the (corrected) positive article yielded a *negative* attitude change in a participant), this number was multiplied by −1. In this way, continued influence was always represented as a positive number, and a reversal of the intended persuasive effect (a “backfire” effect) was always represented as a negative number—regardless of whether the misinformation was positive or negative. Phrased differently, the persuasive impact measure indicates the degree to which participants' attitude changes reflect continued endorsement with the (mis)information they read. There are persuasive impact scores for general impression, and for expected quality of care; scores potentially range from −10 to 10.

#### COVID‐19 overconfidence

A COVID‐19‐related adaptation of the Over‐Claiming Questionnaire (OCQ) of Paulhus and Bruce (1990) was used to measure participants' levels of COVID‐19 overconfidence. Participants indicated how familiar they were with 25 COVID‐19‐related concepts—eight of which vaguely resembled COVID‐related terms, but were non‐existent (e.g., “Meta‐toxides,” “Huwan,” “Viral conjuctivus”). Measurement was on a scale from 1 (*never heard of it*) to 5 (*very familiar*). Higher scores on the non‐existent phenomena indicate overconfidence (*M* = 2.19, *SD* = 0.62; ω = 0.67, 95% CI [.62, .72]).

Materials can be found in the Supporting Information Study 1, as well as details about factor analyses for all scales.

### Results

#### Manipulation checks

An independent t‐test showed that the manipulation of article valence was successful (*t*(401) = −42.38, *p* < .001, *d* = 4.29). Participants perceived the positive article as more positive (*M* = 3.52, *SD* = 1.48) than the negative article (*M* = ‐3.13, *SD* = 1.62). The correction manipulation also succeeded (*t*(401) = 2.15, *p* = .032, *d* = 0.22). Participants perceived the corrected misinformation as less true (*M* = −0.18, *SD* = 2.13) than the uncorrected misinformation (*M* = 0.28, *SD* = 2.11).

#### Effects of corrections and article valence on persuasive impact

To test whether COVID‐19‐related misinformation continues to have persuasive impact after correction (H1), we ran two one‐sample t‐tests on participants who saw a correction. Results show that the persuasive impact of COVID‐19‐related misinformation did not significantly differ from 0 for both general impression of the hospital (*M* = −0.01, *SD* = 2.00, *t*(204) = −0.70, *p* = .944), and expected quality of care (*M* = 0.18, *SD* = 1.97, *t*(204) = 1.28, *p* = .203). H1 thus was rejected: on average, no continued influence of misinformation remained after correction.^3^

To determine whether continued influence was stronger for negative than for positive misinformation (H2), we conducted two independent t‐tests on participants who saw a correction. Indeed, we found a difference between persuasive impact of the corrected negative (*M* = 0.47, *SD* = 1.82) and positive misinformation (*M* = −0.53, *SD* = 2.06; *t*(203) = 3.67, *p* < .001, *d* = .51) for general impression, and also for expected quality of care (negative: *M* = 0.88, *SD* = 1.85 vs. positive: *M* = −0.58, *SD* = 1.81; *t*(203) = 5.67, *p* < .001, *d* = .80). Notably, the persuasive impact (or: endorsement) of the corrected negative misinformation is significantly above zero (one‐sample *t*‐test: *p* = .013 and *p* = .002, respectively), indicating continued influence, while the persuasive impact of the corrected positive misinformation is now significantly below zero (*p* = .009 and *p* < .001, respectively), indicating a backfire effect. These results confirm H2: continued influence is indeed stronger for negative misinformation than for positive misinformation. In fact, continued influence *only* applies to corrected negative misinformation; corrections of positive misinformation result in a significant backfire effect.^3^

**Figure 1:**
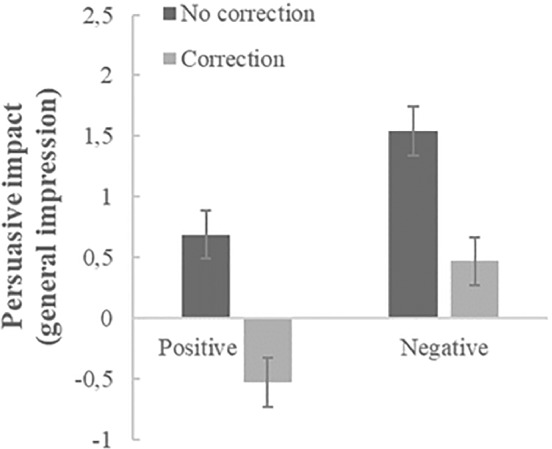
Mean persuasive impact (/endorsement) of non‐corrected versus corrected positive and negative misinformation on general impression of the hospital. Error bars denote *SE* around the mean.

**Figure 2:**
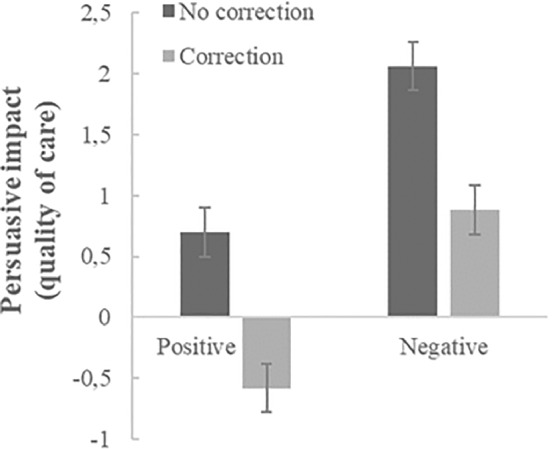
Mean persuasive impact (/endorsement) of non‐corrected versus corrected positive and negative misinformation on expected quality of care. Error bars denote *SE* around the mean.

To check whether the persuasive effects of the corrected misinformation differ from those of their uncorrected counterparts, we conducted two valence*correction ANOVA's—this time including participants who did not see a correction (see Figures 1 and 2 for condition means). Significant main effects of correcting, *F*(1,399) = 34.24, *p* < .001, *η*_p_^2^ = .079, and valence of the article, *F*(1,399) = 22.41, *p* < .001, *η*_p_^2^ = .053, were found for the persuasive impact of misinformation on impression of the hospital (see Table S1 in Supporting Information Study 1, for means for the main effects). More interestingly, no interaction effect between correction and valence was found (*F*(1,399) = 0.15, *p* = .702, *η*_p_^2^ < .001), indicating that the corrections reduced the persuasive impact/endorsement of the positive and negative misinformation to a similar extent. The same pattern was found for expected quality of care: significant main effects for correction (*F*(1,399) = 38.67, *p* < .001, *η*_p_^2^ = .088) and valence (*F*(1,399) = 50.69, *p* < .001, *η*_p_^2^ = .113), but no interaction between correction and valence (*F*(1,399) = 0.51, *p* = .822, *η*_p_^2^ < .001).

In sum, our data show (as displayed in Figures 1 and 2) that corrections reduce the persuasive impact/endorsement of misinformation; they also show—in line with our expectations—that negative misinformation remains persuasive while the endorsement of the positive misinformation reverses to opposition after correction.

#### Effects of overconfidence on continued influence

We conducted two regression analyses testing the effect of overconfidence on continued influence (H3), again only including participants who saw a correction. Contrary to our expectations, overconfidence did not predict persuasive impact on general impression (*p* = .95), nor on expected quality of care (*p* = .77; see Table S2 in Supporting Information Study 1, for details). Hence, H3 is rejected: news consumers' overconfidence does not induce continued influence effects. Following H4, we expected a two‐way interaction between overconfidence and valence on persuasive impact. This effect was neither found for general impression (*p* = .706) nor for quality of care (*p* = .738; see Table S3 in Supporting Information Study 1). Hence, H4 is also rejected. Overall, our results suggest that overconfidence does not affect the persuasive impact of misinformation.

### Discussion

Study 1 suggests that corrections are effective in countering persuasive effects of positive misinformation, but that for negative misinformation, intended persuasive effects persist. These results align with prior studies (Guillory & Geraci, 2016; Lewandowsky et al., 2012), and with Hypothesis 2. The expectation that continued influence of misinformation would generally occur (Hypothesis 1), however, was not confirmed.

The finding that continued influence effects occur for negative but not for positive misinformation, does not necessarily imply that corrections for negative misinformation are less effective. In fact, our analyses suggest that corrections reduced the persuasive impact of positive and negative messages to an equal extent. We do find—congruent with Baumeister et al. (2001)—that negative information more strongly affects attitudes to begin with. Corrections simply cannot entirely eliminate this influence.

In contrast to other studies (Motta et al., 2018; Pennycook & Rand, 2020), we did not observe a significant role for overconfidence in explaining continued influence effects.

### Limitations

Possible limitations of the current study include the use of a convenience sample, which may explain the relative high education level of the participants. This may potentially have reduced persuasive effects of misinformation. Second, with hindsight, participant inclusion criteria (18+, and between 5 and 40 minutes of test duration) should have been pre‐registered. Third, thanks to the reviewers, we became aware of some small inconsistencies in the materials that may have affected participant responses. While the positive news article discussed, apart from recovery rates, the treatment of Dutch patients, the negative article discussed the lack of ICU beds. Similarly, while the correction for the positive article discussed the hospital's evaluation and the treatment of Dutch patients, the correction for the negative article only discussed the former. Fourth, relatedly, the two news articles may have differed in plausibility; it might be perceived unlikely by Dutch participants that a German hospital was the worst in Western Europe. This could have reduced the negative article's persuasive impact, in turn reducing the effect found for H2. Fifth and finally, participants closer to the German border may have been more familiar with German health care (however, note that the hospitals used were fictional). To address these issues and reconfirm Study 1's results, we replicated it with targeted modifications.

## STUDY 2

The design of Study 2 emulates that of Study 1. The pre‐registration document, including inclusion criteria, can be found on the OSF (https://osf.io/jsu24/?view_only=8028d4a1194e4787a9187d4f49de8f33). The study was also checked for compliance to the home institute's ethical guidelines.

### Participants

Power analyses suggested a required sample size of 400 (see Study 1). To account for pre‐registered exclusion of overly fast (<2 minutes) or slow (>30 minutes) participants, we oversampled by 10%. Hence, 440 participants were recruited, through Prolific.co. Participants received £0.95 for a 7‐minute questionnaire (£8.14 p/h). To avoid systematic variations in familiarity with the German health‐care system, only UK residents were selected. Three participants were deleted because they exceeded the 30‐minute time limit, leaving 437 participants. Average age: 35.9 years; range 18–76, *SD* = 13.7; 65.9% female; 57.0% had a bachelor degree or higher; 94.3% had the British nationality.

### Stimuli

The stimuli were similar to those used in Study 1 (for details, see Supporting Information Study 2). Key differences are that (a) the hospitals were presented as performing best (*N* = 218) versus worst (*N* = 219) *within Germany* (i.e., not within Western Europe), thus avoiding the potential problem of asymmetric plausibility, (b) both articles focused on COVID‐19 recovery/mortality rates, and patient care, and (c) both corrections stated that (i) recovery/mortality rates were similar to other hospitals, and (ii) other claims in the article were unsubstantiated.

### Procedure and measures

Procedures and measures emulated Study 1. Two measures (state‐anxiety and hospital reputation), were omitted for reasons of brevity and redundancy with respect to the tested hypotheses. COVID‐19 overconfidence was measured more to the letter of the original (see also, Pennycook & Rand, 2020) by including only three non‐existent items between 12 existing ones (*M* = 1.22, *SD* = 0.46; *r* = .30, *p* < .01 after removing one item). Persuasive impact and COVID‐concern (*M* = 3.41, *SD* = 0.94; *α* = .85) were measured equally to Study 1.

### Results

#### Manipulation checks

Manipulation checks were successful: the positive article was perceived as more positive (*M* = 4.36, *SD* = 1.06) than the negative article (*M* = −3.93, *SD* = 1.57; *t*(435) = −64.62, *p* < .001, *d* = 6.19), and the corrected information as less true (*M* = −1.43, *SD* = 2.57) than the not corrected information (*M* = 1.76, *SD* = 1.88; *t*(435) = 14.84, *p* < .001, *d* = 1.42).

#### Effects of valence and corrections on persuasive impact

One‐sample t‐tests on the participants who saw a correction showed that the persuasive impact of corrected misinformation did not significantly differ from 0 for both the general impression of the hospital (*M* = 0.01, *SD* = 2.28, *t*(215) = 0.09, *p* = .929), and the expected quality of care (*M* = 0.15, *SD* = 2.43, *t*(215) = 0.90, *p* = .372). Thus, like in Study 1, H1 was rejected; the corrections neutralised the persuasive effects (or, endorsement) of the misinformation to a value near zero.

Testing H2, two independent t‐tests conducted on participants who saw a correction, showed that for general impression, continued influence for corrected negative misinformation (*M* = 0.82, *SD* = 1.65) was higher than for corrected positive misinformation (*M* = −0.80, *SD* = 2.52; *t*(214) = 5.58, *p* < .001, *d* = .76). The same results were found for expected quality of care (positive: *M* = −0.95, *SD* = 2.28 vs. negative: *M* = 1.25, *SD* = 2.06; *t*(214) = 7.46, *p* < .001, *d* = 1.01). Hence, like in Study 1, H2 was confirmed. Notably, both the continued influence of corrected negative misinformation and the backfire effect of positive misinformation were significantly different from zero (all *p*'s < .002).

**Figure 3:**
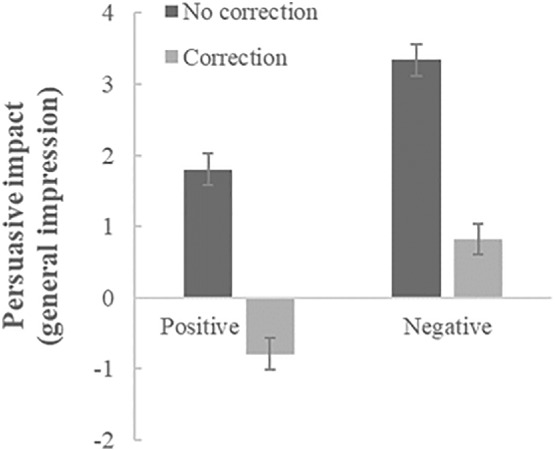
Mean persuasive impact (/endorsement) of non‐corrected versus corrected positive and negative misinformation on general impression of the hospital. Error bars denote *SE* around the mean.

**Figure 4:**
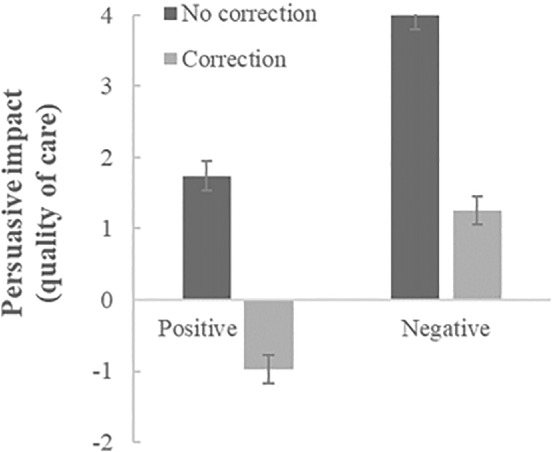
Mean persuasive impact (/endorsement) of non‐corrected versus corrected positive and negative misinformation on expected quality of care. Error bars denote *SE* around the mean.

The correction*valence interaction analyses conducted on all participants (see Figures 3 and 4 for condition means) also showed the same effects as Study 1: A significant main effect of correction (*F*(1,433) = 137.25, *p* < .001, *η*_p_^2^ = .241) and of article valence (*F*(1,433) = 52.48, *p* < .001, *η*_p_^2^ = .108) on general impression, but no interaction effect (*F*(1,433) = 0.03, *p* = .858, *η*_p_^2^ < .001). Similar for expected quality of care: main effects of correction (*F*(1,433) = 150.51, *p* < .001, *η*_p_^2^ = .258) and article valence (*F*(1,433) = 101.42, *p* < .001, *η*_p_^2^ = .190), but no interaction effect (*F*(1,433) = 0.13, *p* = .908, *η*_p_^2^ < .001). The absence of interaction effects indicates that the corrections for the positive and negative misinformation were equally effective (means for main effects in Table S4 in Supporting Information Study 2).

In sum, like in Study 1, our analyses show (see, again, Figures 3 and 4) that corrections reduce the persuasiveness/endorsement of the original misinformation, but that negative misinformation continues to be persuasive while the positive misinformation backfires.

#### The effects of overconfidence on continued influence

Contrary to H3, overconfidence did not predict persuasive impact of corrected misinformation on general impression (*p* = .94), nor on expected quality of care (*p* = .71). Contrary to H4, there was no two‐way interaction between overconfidence and valence on persuasive impact for general impression (*p* = .75) nor for quality of care (*p* = .57). These findings all follow Study 1.

### Discussion

Study 2—conducted with UK residents instead of Dutch, and using modified materials to avoid confounds—produced exactly the same results as Study 1. Again, continued influence effects are only found for negative misinformation. For positive misinformation, the correction causes a backfire effect, resulting in reversed receiver attitudes. Possibly due to the improved stimuli, the effects in Study 2 are more pronounced than in Study 1. Also similar to Study 1, overconfidence did not affect continued influence effects.

## GENERAL DISCUSSION

The present studies focused on the persuasive impact of COVID‐19‐related misinformation and the moderating effects of features of misinformation itself and those of the recipient of the information. In line with studies that show that corrections often fail in their effectiveness (e.g., Johnson & Seifert, 1994), we expected misinformation to have continued influence (i.e., remaining persuasive impact in the intended direction) even after correction. Our results suggest that corrections are effective in countering the persuasive effects of positive misinformation, but that in the case of negative misinformation, intended persuasive effects persist.

Although these results are in line with our second hypothesis and with prior studies showing continued influence effects for negative information (Lewandowsky et al., 2012), they do not support our first hypothesis, in which we expected to find a continued influence effect for corrected misinformation in general. What we did find is that continued influence effects *only* applied to negative misinformation, and *not* to positive misinformation. This is in line with the findings by Guillory and Geraci (2016), who show that the reliance on (political) positive misinformation is reduced to zero after correction, whereas the reliance on corrected negative misinformation is only partly reduced. We partly replicate these findings in a health context, but add to them that for corrected positive misinformation the persuasive effects reverses: after correction of positive misinformation people perceive the hospital more negatively than they did before they ever read anything about it. Interestingly, this “backfire” effect of corrected positive information was previously observed in political misinformation (Cobb et al., 2013), but our results suggest it also applies to health misinformation.

Although we found continued influence effects for negative misinformation to be stronger than for positive misinformation, this does not mean that corrections of negative misinformation are less effective. When we look at the condition means, we see that the corrections reduced the persuasive impact of the positive and negative messages to an equal extent (hence, no interaction effects between fact‐check and valence were observed). These results imply that the asymmetry in continued influence is not caused by differential effectiveness of the corrections, but rather by a difference in the initial persuasive impact of the positive versus negative misinformation. This observation is in line with the previously mentioned negativity bias (Baumeister et al., 2001), which states that negative information tends to outweigh positive information. From an evolutionary perspective (e.g., Vaish et al., 2008), it makes sense that people attribute more weight to negative information than to positive information, as the former may assist in avoiding severe and potentially irreversible harm. Our results congruently suggest that negative information is harder to correct because it had more influence in the first place.

Overconfidence does not seem to play a significant role in continued influence of misinformation. This contradicts previous studies, where a clear link between knowledge overestimation and belief in misinformation was found (Motta et al., 2018; Pennycook & Rand, 2020). A possible explanation for our findings might be that we used a specific COVID‐19 overclaiming measure, while other studies often use a general overclaiming measure focusing on different kinds of knowledge—rendering overconfidence a personality, rather than a contextual, variable. Further studies could investigate if possibly general knowledge overclaiming may predict continued influence of health‐related misinformation.

A possible limitation of the current studies might be the participants' relatively high education levels. High levels of education may make individuals less inclined to be persuaded by misinformation. Moreover, individuals high in cognitive ability are more inclined to update mental models after misinformation is corrected (De Keersmaecker & Roets, 2017). This could possibly weaken persuasive effects of misinformation and strengthen those of the correction. If so, the make‐up of our samples may have led us to underestimate continued influence effects. We might speculate that backfire effects are also less pronounced in high ability individuals, but as far as we know, this issue has not been empirically addressed.

Another possible limitation of our study is in the instruments we used to measure overconfidence. Some of the overclaiming items used in Study 1 might have looked familiar to participants (e.g., Huwan instead of Wuhan), causing them to “recognise” the term, while this false recognition actually might be prompted by cognitive ease. We tried to address this issue by altering the measure for Study 2; however, this time internal consistency was low (one of the three items needed to be removed). We would urge future researchers of the relationship between overconfidence and susceptibility to misinformation to first focus on constructing a valid measure of (contextual) overconfidence.

A third limitation of our study could be in the COVID‐19‐related misinformation we presented to participants. Remember that we found it ethically irresponsible to predict continued influence of misinformation on the one hand, and to distribute potentially noxious misinformation on the other. Therefore, we chose to use misinformation that was benign both in nature and personal relevance. One could wonder whether more serious and personally relevant misinformation would have yielded different effects. On the one hand, personal relevance of misinformation may evoke more analytic processing, possibly reducing continued influence effects. On the other hand, the fear that serious and relevant misinformation may induce, may evoke superficial processing. Additionally, a lower psychological distance (e.g., for more personal relevant, proximal information) potentially increases biased information processing (Bates & Peynircioğlu, 2017). Perhaps future researchers will be able to solve the ethical Catch‐22, and study continued influence effects using more serious misinformation.

With the current study, we contribute to research focusing on misinformation effects in general, and in the COVID‐19 realm in particular. Our results indicate that it is advisable to correct misinformation, because even though corrections might not completely neutralise the impact of misinformation, it is at least reduced. Nevertheless, results also suggest that especially negative misinformation may have lasting impact. Since the spread of (online) misinformation is currently a serious problem, it would be useful to train online news consumers to process information in a more critical way, for example, by verifying the news source before consuming the news. Such strategies can make news consumers, and society, more resilient to the negative impact that misinformation can have on health.

In conclusion, our study shows that COVID‐19 related misinformation may still have continued influence after correction, but only in the case of negative misinformation. Correction of positive misinformation showed to be effective and yields an attitude reversal, or “backfire” effect, rather than continued influence. In times of crisis, such as during the COVID‐19 pandemic, misinformation almost spreads as fast the virus itself. Our study shows that neutralising the persuasive impact of negative misinformation may be difficult. Public awareness of continued influence effects and of individual strategies to prevent persuasive impact of misinformation, may help to keep misinformation from becoming a public health threat.

## ETHICAL COMPLIANCE

All procedures performed in this study were in accordance with the ethical standards of the institutional research committee and with the 1964 Helsinki Declaration and its later amendments or comparable ethical standards. Informed consent was obtained from all individual participants included in the study.

## Supporting information


**Appendix S1.** Supporting information.

**Appendix S2.** Supporting information.

## ENDNOTES

^1^
Note that we did not state a 2x2 interaction hypothesis, because we did not expect corrections to be more effective for positive or negative misinformation. To achieve .8 power for such a 2x2 interaction, we would have required a sample size of around 500 (Perugini et al., 2018).

^2^
Analyses of these variables can be found in the Supporting Information Study 1, Appendix J1.

^3^
For reasons of robustness, we re‐tested H1 and H2 including all complete data records—that is, including the fast and slow and young participants. H1 was still rejected: no significant continued influence remained for general impression (*t*(220) = 0.00, *p* = 1.00), and expected quality of care (*t*(220) = 1.49, *p* = .138). H2 was still accepted: we found a significant difference between persuasive impact of the and the positive corrected misinformation for both general impression *(t*(219) = 4.16, *p* < .001) and expected quality of care *t*(219) = 5.95, *p* < .001).
