# Supplementary material for: Continued influence of misinformation in times of COVID‐19
Source: Int J Psychol. 2021 Aug 26;57(1):136–45. doi: 10.1002/ijop.12805 (PMC8652781; doi:10.1002/ijop.12805)
Supplement: Supplementary file 1 — Appendix S1. Supporting information. [file IJOP-57-136-s002.pdf]

Supporting Information Study 1

Supporting Information Legend

Appendix A1

1. Original stimuli presented in the positive valence condition: positive misinformation article.
2. Translated stimuli of the positive valence condition.
3. Original Stimuli presented in the negative valence condition: negative misinformation article.
4. Translated stimuli of the negative valence condition.

Appendix B2

1. Original stimuli presented in the positive valence, debunking fact-check condition: debunking fact-check positive misinformation.
2. Translated stimuli of the debunking fact-check in the positive valence condition.
3. Original stimuli presented in the negative valence, debunking fact-check condition: debunking fact-check negative misinformation.
4. Translated stimuli of the debunking fact-check in the negative valence condition.

Appendix C3

All five images of fictional hospitals used in the evaluation task at both T1 (before the misinformation article was presented) and T2 (after the misinformation article was presented, and if applicable, debunked).

Appendix D1

1. Original Dutch version of the COVID-19 Over-Claiming Questionnaire.
2. English translation of the COVID-19 Over-Claiming Questionnaire.

Appendix E1

1. Original Dutch adaptation of the Customer-Based Corporate Reputation Scale.
2. English translation of the Adaptation of the Customer-Based Corporate Reputation Scale.

Appendix F1

1. Original Dutch 6-item short-form State Scale.
2. Original Dutch 4-item COVID Concern Questionnaire.
3. English translation of the 6-item short-form State Scale.
4. English translation of the 4-item COVID Concern Questionnaire.

Appendix G1

Table S1: Means and standard deviations of the two 2x2 ANOVAs with persuasive impact of misinformation on impression and expected quality of care of the hospital as dependent variables.

Appendix H1

Table S2: Results of a linear regression analysis with the persuasive impact of misinformation on impression and expected quality of care of the hospital as dependent variables.

Table S3: Results of a multiple linear regression analysis with the persuasive impact of misinformation on impression and expected quality of care of the hospital as dependent variables.

Appendix I1

Measurement details and factor analyses of the measurements: Over-Claiming Questionnaire, State anxiety, and COVID-19 threat.

Appendix J1

Analyses extra variables: State anxiety, COVID-19 threat, Hospital reputation.

Appendix A1

1. Original Stimuli presented in the positive valence condition: positive misinformation article.

NL Dagelijks dagblad

Ongekende Zorg in Tijden van Corona: Städtisches Klinikum Düsseldorf door HealthRank Verkozen tot Beste West-Europese Ziekenhuis

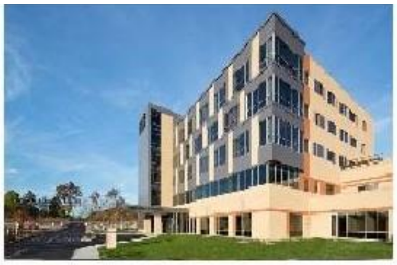

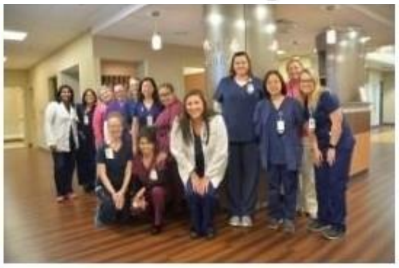

Gisteren werden de jaarlijkse HealthRank Awards uitgereikt, die ditmaal bijna geheel in het teken stonden van de Coronacrisis. Het Duitse ziekenhuis Städtisches Klinikum Düsseldorf ontving de belangrijkste prijs: De Onderscheiding voor Klinische Excellentie.

De prijzen die HealthRank uitreikt, zijn onder andere gebaseerd op stemmen van patiënten. Zo werd het ziekenhuis beoordeeld als 'een prettige plek om te verblijven' en benoemden veel patiënten 'de goede persoonlijke aandacht.'

Bij de beoordeling werd ook meegenomen wat de aanpak van de ziekenhuizen was betreffende het Coronavirus. Het Städtisches Klinikum Düsseldorf was goed voorbereid en heeft door tekort aan Nederlandse IC-bedden ook veel Nederlandse Coronapatiënten opgevangen. Daarnaast is er in dit ziekenhuis geen enkele patiënt overleden aan COVID-19 en zijn alle IC-patiënten binnen afzienbare tijd hersteld door de goede zorg, waarna ze verder verpleegd werden op reguliere afdelingen.

Directeur Aaron Müller reageerde enthousiast: 'Het is fijn om te zien dat ons ziekenhuis zo goed wordt beoordeeld. Onze patiënten zijn het allerbelangrijkst en het is fijn om te horen dat ze een doorgaans moeilijke tijd in het ziekenhuis toch positief hebben ervaren. Wij blijven hard werken om de zorg voor onze patiënten net zo goed te houden of zelfs te verbeteren!'

30-04-2020 ©

2. Translated stimuli of the positive valence condition

NL Daily paper

Unprecedented Care in Times of Corona: Städtisches Klinikum Düsseldorf Named Best Western European Hospital by HealthRank

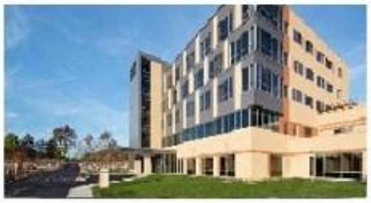

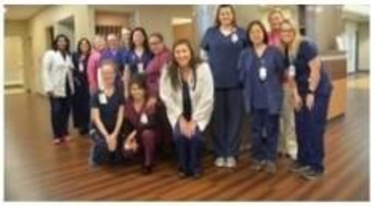

The annual HealthRank Awards ceremony took place yesterday, which almost completely revolved around the Corona crisis. The German hospital Städtisches Klinikum Düsseldorf received the most important award: The Award for Clinical Excellence.
The awards provided by HealthRank are partly based on votes of patients. The hospital was named ‘a comfortable place to stay’ and many patients emphasized ‘the nice personal attention’ they received.

Part of the assessment of the hospitals was their mode of operation regarding the Corona virus. The Städtisches Klinikum Düsseldorf was well prepared and accommodated numerous Dutch COVID-19 patients, who could not be hospitalized in their own country due to a lack of ICU capacity. What’s more is that not a single COVID-19 patient of the Städtisches Klinikum Düsseldorf passed away, and that all COVID-19 ICU patients recovered within a relatively short time, where after they were moved to regular hospital wards where they received further treatment if necessary.

Managing director Aaron Müller responded enthusiastically: ‘It’s so nice to see that our hospital received such a wonderful evaluation. Our patients are what’s most important to us, and it’s great to hear they experienced this usually difficult time in a hospital quite positively anyway. We will continue our work providing great care for our patients and we will even try to improve it!’

30-04-2020 ©

3. Original Stimuli presented in the negative valence condition: negative misinformation article.

NL Dagelijks dagblad

Zorg in Tijden van Corona: Hoogste Sterftecijfer onder Patiënten in het Duitse Ziekenhuis Städtisches Klinikum Düsseldorf

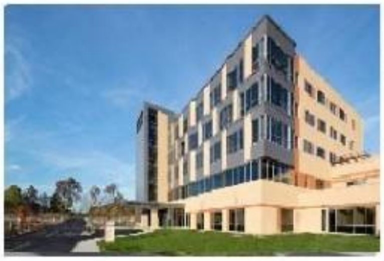

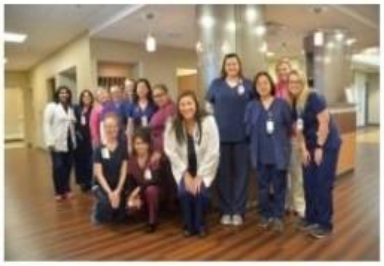

Gisteren publiceerde de organisatie HealthRank de medische cijfers van alle West-Europese ziekenhuizen. Uit deze cijfers blijkt dat het Duitse ziekenhuis Städtisches Klinikum Düsseldorf het slechtst presteert.

Patiënten laten weten dat het ziekenhuis een 'onprettige plek is om te moeten verblijven' en velen noemden 'het gebrek aan persoonlijke aandacht.' Het ziekenhuis kampt ook met enorme wachtlijsten voor standaardingrepen. Daarnaast bleken met name de Coronapatiënten die op de Intensive Care hadden gelegen ontevreden te zijn. Voormalig patiënt Aaron Müller vertelt: 'Wanneer je ontwaakt uit een kunstmatige coma heb je zowel psychische als fysieke zorg nodig. Ik mocht mijn familie of geliefden niet zien en niemand vertelde me waar ik was of wat er met me aan de hand was. Daarnaast was al het zorgpersoneel natuurlijk gekleed in speciale pakken, waardoor ik geen enkele dokter herkende. Het voelde allemaal erg eenzaam en bedreigend.'

Bij de beoordeling werd ook meegenomen wat de aanpak van de ziekenhuizen was betreffende het Coronavirus. Het Städtisches Klinikum Düsseldorf bleek niet goed voorbereid te zijn en er bleek een tekort te zijn aan IC-bedden. Hierdoor konden niet alle patiënten worden opgevangen. Daarnaast blijkt een schrikbarend deel van de patiënten te zijn overleden: ruim 85% van de IC-patiënten zijn overleden aan de gevolgen van COVID-19 sinds 16 maart 2020. Ook het algemene sterftecijfer van het Städtisches Klinikum Düsseldorf ligt 23% hoger dan het gemiddelde sterftecijfer van andere West-Europese ziekenhuizen.

30-04-2020 ©

4. Translated stimuli of the negative valence condition.

NL Dagelijks dagblad

Care in Times of Corona: Highest Mortality Rates in German Hospital Städtisches Klinikum Düsseldorf

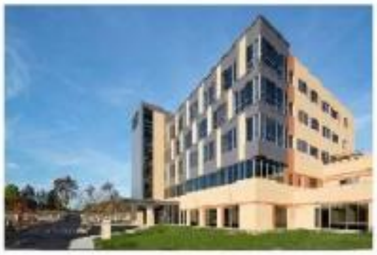

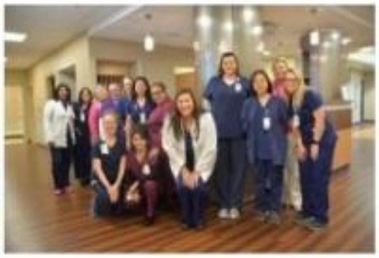

Yesterday, HealthRank published the medical figures of all Western European hospitals. These figures show that the German hospital Städtisches Klinikum Düsseldorf shows the worst performance.

Patients report that the hospital is an "unpleasant place to stay," and many cited "the lack of personal attention." The hospital also faces huge waiting lists for standard surgery. In addition, in particular Corona patients who had been in the Intensive Care Unit turned out to be dissatisfied. Former patient Aaron Müller says: "When you wake up from an artificial coma, you need both psychological and physical care. I was not allowed to see my family or loved ones, and no one told me where I was or what was wrong with me. In addition, all healthcare personnel were of course dressed in special suits, so I did not recognize a single doctor. It all felt very lonely and threatening."

The assessment also included the approach taken by the hospitals regarding the Corona virus. The Städtisches Klinikum Düsseldorf turned out to be ill-prepared, and suffered from a shortage of IC beds. As a result, not all patients could be treated. In addition, an alarming number of patients appears to have passed away: more than 85% of IC patients have died from the consequences of COVID-19 since March 16, 2020. The general mortality rate at the Städtisches Klinikum

30-04-2020 ©

Appendix B1

1. Original stimuli presented in the positive valence, debunking fact-check condition:

   debunking fact-check positive misinformation

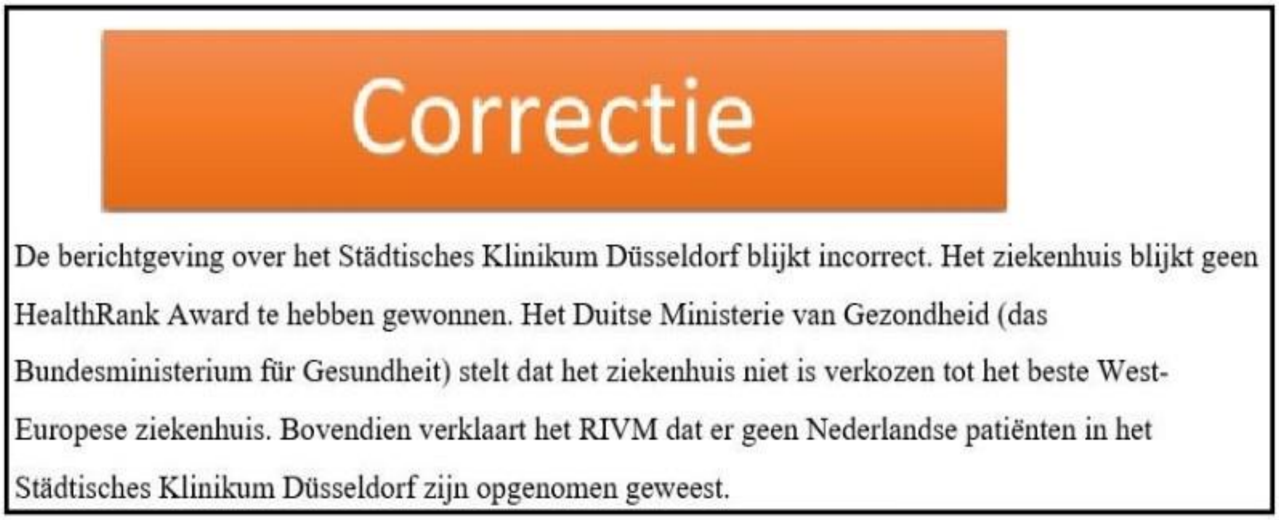

Correctie

De berichtgeving over het Städtisches Klinikum Düsseldorf blijkt incorrect. Het ziekenhuis blijkt geen HealthRank Award te hebben gewonnen. Het Duitse Ministerie van Gezondheid (das Bundesministerium für Gesundheit) stelt dat het ziekenhuis niet is verkozen tot het beste West-Europese ziekenhuis. Bovendien verklaart het RIVM dat er geen Nederlandse patiënten in het Städtisches Klinikum Düsseldorf zijn opgenomen geweest.

2. Translated stimuli of the debunking fact-check in the positive valence condition.

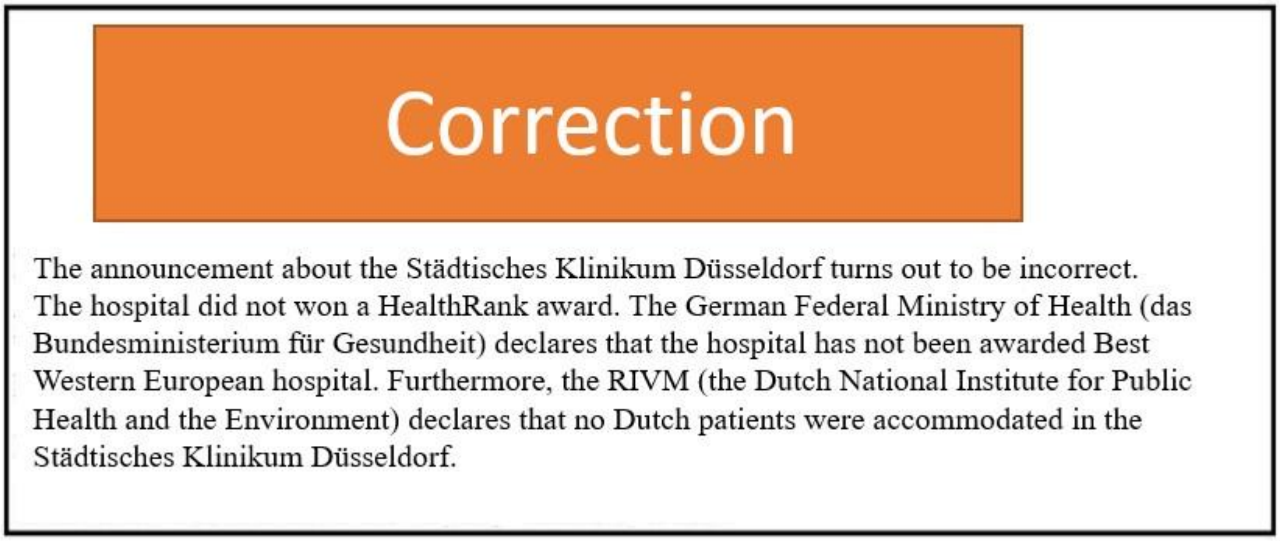

Correction

The announcement about the Städtisches Klinikum Düsseldorf turns out to be incorrect. The hospital did not won a HealthRank award. The German Federal Ministry of Health (das Bundesministerium für Gesundheit) declares that the hospital has not been awarded Best Western European hospital. Furthermore, the RIVM (the Dutch National Institute for Public Health and the Environment) declares that no Dutch patients were accommodated in the Städtisches Klinikum Düsseldorf.

3. Original stimuli presented in the negative valence, debunking fact-check condition: debunking fact-check negative misinformation.

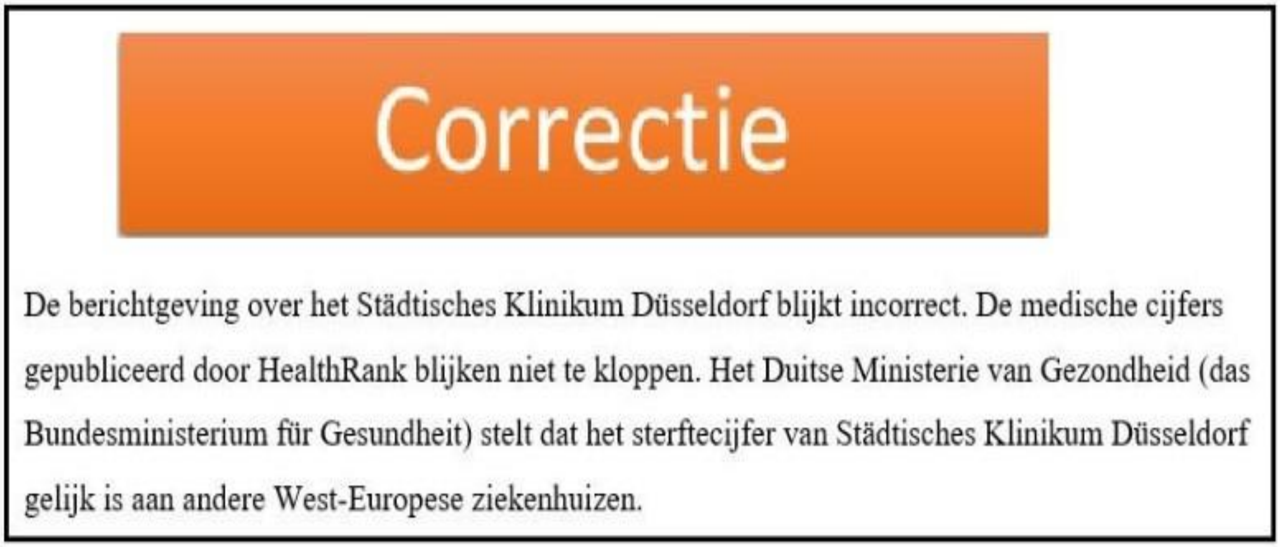

Correctie

De berichtgeving over het Städtisches Klinikum Düsseldorf blijkt incorrect. De medische cijfers gepubliceerd door HealthRank blijken niet te kloppen. Het Duitse Ministerie van Gezondheid (das Bundesministerium für Gesundheit) stelt dat het sterftecijfer van Städtisches Klinikum Düsseldorf gelijk is aan andere West-Europese ziekenhuizen.

4. Translated stimuli of the debunking fact-check in the negative valence condition.

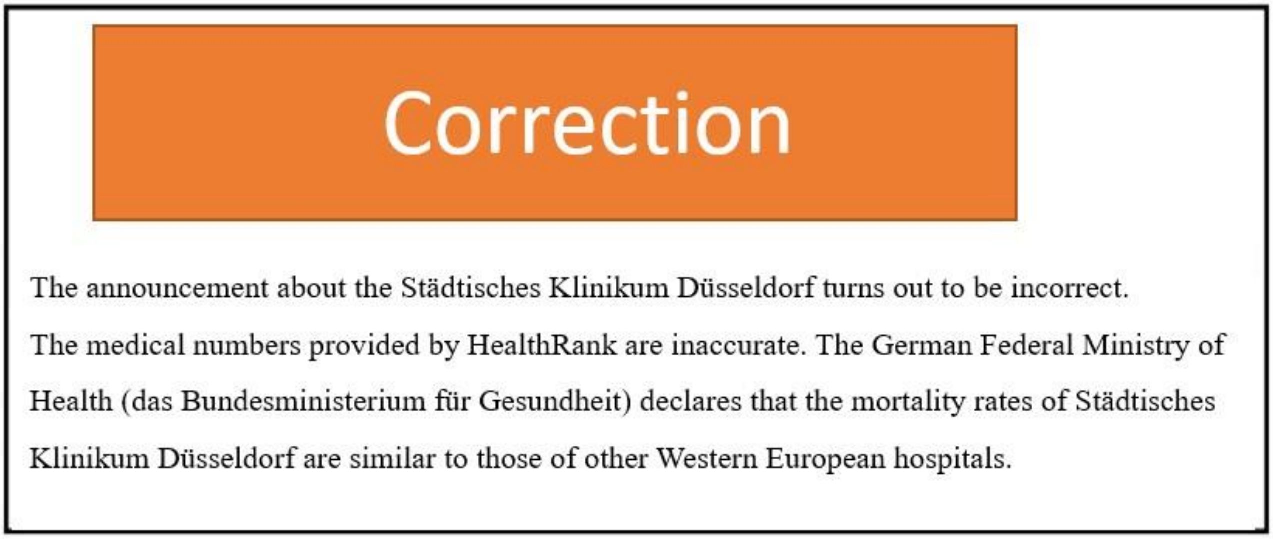

Correction

The announcement about the Städtisches Klinikum Düsseldorf turns out to be incorrect.
The medical numbers provided by HealthRank are inaccurate. The German Federal Ministry of Health (das Bundesministerium für Gesundheit) declares that the mortality rates of Städtisches Klinikum Düsseldorf are similar to those of other Western European hospitals.

Appendix C1

All five images of fictional hospitals used in the evaluation task as both T1 (before the misinformation article was presented) and T2 (after the misinformation article was presented, and if applicable, debunked).

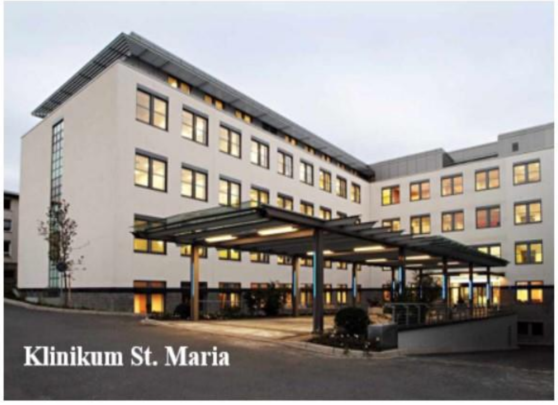

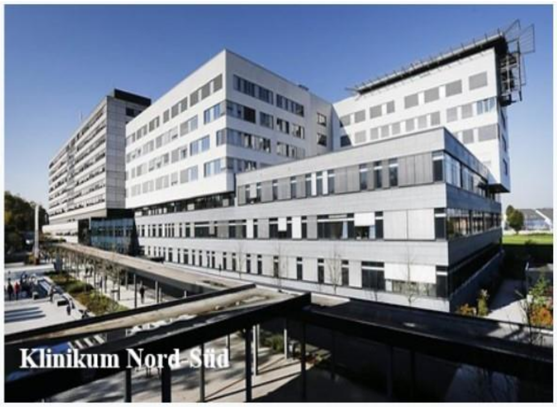

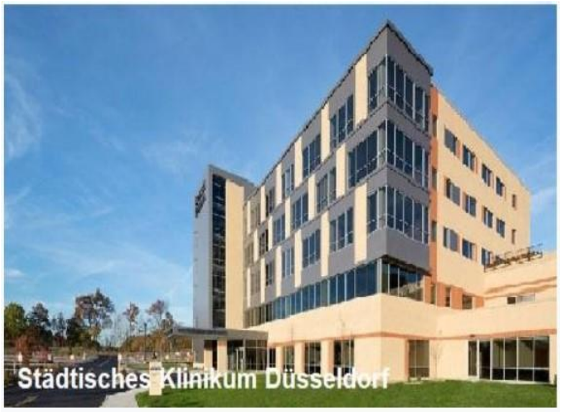

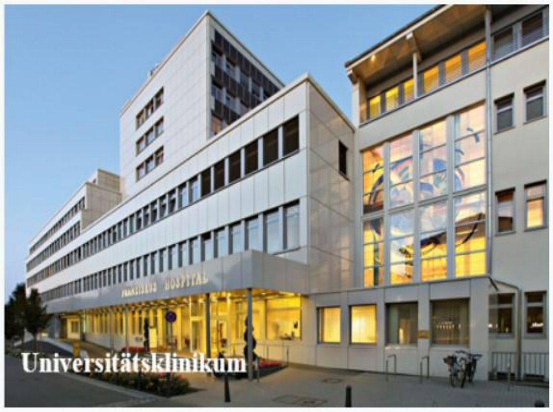

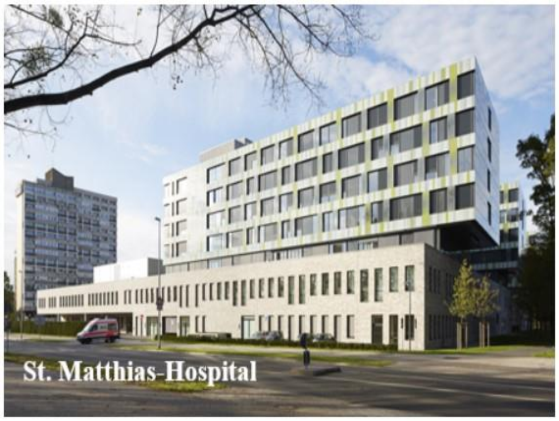

Appendix D1

Dutch COVID-19 Over-Claiming Questionnaire (based on the OCQ; Paulhus & Bruce, 1990).

5-point Likert-scale (never heard of it, vaguely familiar, somewhat familiar, familiar, very familiar)

Zou u bij de volgende vragen kunnen aangeven in hoeverre u bekend bent met de persoon, het ‘ding’, het woord, de plaats, of het idee/concept dat in de vraag genoemd wordt?

Items:

1. Jaap van Dissel
2. Beademingsbol
3. Volksimmuniteit
4. Zoönose
5. Anderhalvemetersamenleving
6. Aerosolinfectie
7. Anthony Fauci
8. Meta-toxides
9. Antroponose
10. Acute Respiratory Distress Syndrome
11. Chloroquine
12. Schubdieren
13. Hydrochlidorine
14. SARS-CoV-2
15. Severe Acute Inhalation Syndrome
16. Outbreak Management Team
17. Postintensivecaresyndroom
18. Huwan
19. Saturatie
20. Virale conjuctivus
21. FSK S1-PHN enzym
22. Groepsimmuniteit
23. Hamsteren
24. Orthocoronavirinae
25. HCoV-NL63-MeRs19

English translation of the COVID-19 Over-Claiming Questionnaire (based on the OCQ; Paulhus & Bruce, 1990).

5-point Likert-scale (never heard of it, vaguely familiar, somewhat familiar, familiar, very familiar

Could you please indicate how familiar you are with the following (historical) people, 'things', words, places, or the idea/concept that is mentioned in the question?

Items:

1. Jaap van Dissel
2. Respiration sphere
3. Nation immunity
4. Zoonosis
5. One-a-half-meter distance society
6. Aerosol infection
7. Anthony Fauci
8. Meta-toxides
9. Anthroponosis
10. Acute Respiratory Distress Syndrome
11. Chloroquine
12. Pangolins
13. Hydrochlidorine
14. SARS-CoV-2
15. Severe Acute Inhalation Syndrome
16. Outbreak Management Team
17. Post-intensive care syndrome
18. Huwan
19. Saturation
20. Viral conjuctivus
21. FSK S1-PHN enzym
22. Group immunity
23. Hoarding
24. Orthocoronavirinae
25. HCoV-NL63-MeRs19

Appendix E1

Dutch Adaptation of the Customer-Based Corporate Reputation Scale (CBR; Walsh, Beatty & Shiu, 2009). 5-point Likert-scale from completely disagree to completely agree.

Nu volgen er nogmaals een aantal stellingen met betrekking tot het ziekenhuis Stästisches Klinikum Düsseldorf. Geef alstublieft aan in hoeverre u het eens bent met de volgende stellingen.

Het Duitse ziekenhuis Städtisches Klinikum Düsseldorf …

1. … heeft medewerkers (artsen en verplegend personeel) die de patiënten goed behandelen.
2. … heeft medewerkers (artsen en verplegend personeel) die waarde hechten aan de behoefte van de patiënten.
3. … heeft medewerkers (artsen en verplegend personeel) die bezorgd zijn om de patiënten.
4. … neemt de kwaliteit en zorg voor de patiënten serieus.
5. … geeft om al haar patiënten.
6. … is een sterk en betrouwbaar ziekenhuis.
7. … ontwikkelt innovatieve zorg voor de patiënten.
8. … biedt diensten en zorg van hoge kwaliteit voor de patiënten.
9. … gaat met hoge mate van verantwoordelijkheid om met de zorg en gegevens van de patiënten.
10. … zou haar winst verminderen om ervoor te zorgen een milieuvriendelijke omgeving te creëren voor de patiënten.

English translation of the Adaptation of the Customer-Based Corporate Reputation Scale (CBR; Walsh, Beatty & Shiu, 2009). 5-point Likert-scale from completely disagree to completely agree.

A few statements about the Städtisches Klinikum Düsseldorf hospital will follow now. Please indicate to what extent you agree with the following statements.

The German hospital Städtisches Klinikum Düsseldorf …

1. … has employees (medical doctors and nursing staff) who treat their patients courteously.
2. … has employees (medical doctors and nursing staff) who are concerned about patient needs.
3. … has employees (medical doctors and nursing staff) who are concerned about their patients.
4. … takes quality and care of its patients seriously.
5. … seems to care about all its patients.
6. … is a strong and reliable hospital.
7. … develops innovative care for its patients.
8. … offers high quality services and care to its patients.
9. … takes responsible actions regarding care and information of its patients..
10. … would reduce its profits to ensure a clean environment for its patients.

Appendix F1

State anxiety and COVID-19-related anxiety measures

Dutch 6-item short-form State Scale (Marteau & Bekker, 1992; derived from the Spielberger State-Trait Anxiety Inventory), with an additional general anxiety question.

5-point Likert-scale from completely disagree to completely agree.

Nu volgen er een aantal stellingen met betrekking tot uw gemoedstoestand op dit moment. Geef alstublieft aan in hoeverre u het eens bent met de volgende stellingen.

1. Ik voel me kalm.
2. Ik ben gespannen.
3. Ik ben van streek.
4. Ik ben ontspannen.
5. Ik voel me tevreden.
6. Ik maak me zorgen.
7. Ik voel me angstig.

4 items in Dutch of the COVID Concern Questionnaire (Conway, Woodard & Zubrod, 2020).

5-point Likert-scale from completely disagree to completely agree.

Nu volgen er een aantal stellingen met betrekking tot uw gemoedstoestand op dit moment tegenover het Coronavirus. Geef alstublieft aan in hoeverre u het eens bent met de volgende stellingen.

1. Als ik aan het coronavirus (COVID-19) denk, voel ik me bedreigd.
2. Ik ben bang voor het coronavirus (COVID-19).
3. Ik maak me geen zorgen over het coronavirus (COVID-19)
4. Ik ben bang dat ik of mensen van wie ik hou ziek zullen worden van het coronavirus (COVID-19).

English translation of the 6-item short-form State Scale (Marteau & Bekker, 1992; derived from the Spielberger State-Trait Anxiety Inventory, Spielberger & Gorsuch, 1983), with an additional general anxiety question.

5-point Likert-scale from completely disagree to completely agree.

A number of statements about your feelings will follow now. Please indicate to what extent you agree with the follow statements about how you feel right now, at this moment.

1. I feel calm.
2. I feel tense.
3. I feel upset.
4. I feel relaxed.
5. I feel content.
6. I feel worried.
7. I feel anxious.

English translation of the 4 items of the COVID Concern Questionnaire (Conway, Woodard & Zubrod, 2020).

5-point Likert-scale from completely disagree to completely agree.

Some statements about your feelings regarding the coronavirus will follow now. Please indicate to what extent you agree with these statements.

1. Thinking about the coronavirus (COVID-19) makes me feel threatened.
2. I am afraid of the coronavirus (COVID-19).
3. I am not worried about the coronavirus (COVID-19)
4. I am worried that I, or the people I love, will get sick from the coronavirus (COVID-19).

Appendix G1

Table S1

Means and standard deviations of the two 2x2 ANOVAs with persuasive impact of misinformation on impression and expected quality of care of the hospital as dependent variables.

	Persuasive impact			
	Impression		Quality of care	
Condition	*M*	*SD*	*M*	*SD*
Negative article (*n* = 201)	1.00	1.97	1.47	1.98
Positive article (*n* = 202)	0.08	1.99	0.06	1.99
Debunked article (*n* = 205)	-0.03	2.00	0.51	2.00
Non-debunked article (*n* = 198)	1.11	1.97	1.38	1.97

Appendix H1

Supporting Tables Regression Analyses

Table S2

Results of a linear regression analysis with the persuasive impact of misinformation on impression and expected quality of care of the hospital as dependent variables.

		Persuasive impact				
		Impression		Quality of care		
Predictor	*B*	*SE B*	*β*	*B*	*SE B*	*β*
Overconfidence	0.02	0.27	0.01	-0.08	0.26	-0.02
R^2		.00			.00	

Table S3

Results of a multiple linear regression analysis with the persuasive impact of misinformation on impression and expected quality of care of the hospital as dependent variables.

		Persuasive impact				
		Impression		Quality of care		
Predictor	*B*	*SE B*	*β*	*B*	*SE B*	*β*
Overconfidence	0.01	0.26	0.00	-0.10	0.24	-0.03
Valence	-0.50	0.14	-0.25*	-0.73	0.13	-0.37*
Overconfidence x Valence	0.10	0.26	0.03	0.08	0.24	0.02
R^2		.06			.14	

Note. * p < .001.

Appendix I1

Measurement details and factor analyses of the used scales

An individual factor that might explain continued influence effects is anxiety. COVID-19-related (mis)information can be thought of as anxiety inducing—it deals with health threats and provides uncertainty for the future (Farooq et al., 2020). Possibly, elevated anxiety levels elicited by the presented misinformation will prevent recipients from actively processing the subsequent correction. Conversely, misinformation-elicited anxiety may prompt recipients to attend to potential anxiety-relieving information, which may include the subsequent correction.

Since not much is known about the mediating effect of anxiety on continued influence of misinformation, we will merely explore this effect and not state a hypothesis. The following will be our research question: *Does anxiety (in particular, state anxiety and COVID-19 concern) increase the continued influence of COVID-19 misinformation after correction?*

In addition, we tested the effects of misinformation on hospital reputation.

Measurement details

State anxiety. State anxiety was measured using the 6-item short-form State Scale, adapted by Marteau and Bekker (1992), derived from the Spielberger State-Trait Anxiety Inventory (STAI). Next to the 6 originals statements, we added a direct statement ("I feel scared"). Participants were asked to indicate to what extent they agreed, ranging from 1 (*completely disagree*) tot 5 (*completely agree; M* = 2.14, *SD* = 0.68, ω = .85, 95% CI [.83, .87]).

COVID-19 concern. In order to measure specific COVID-19-related anxiety, four items of the COVID Concern Questionnaire of Conway, Woodard and Zubrod (2020) were used. An example item is "I am worried that I or people I love will get sick from the Coronavirus (COVID-

19)”. Again, 5-point agreement Likert-scale was used (*M* = 2.79, *SD* = 0.82, ω = .80, 95% CI [.75, .83]).

Hospital reputation. Hospital reputation was measured as an additional variable, but was not included in any of the pre-registered hypotheses. Ten items of the Customer-Based Corporate Reputation Scale (Walsh et al., 2009) were adapted to the context of the current study, e.g., “Städtisches Klinikum Düsseldorf is a strong and reliable hospital”; 1 (*completely disagree*) to 5 (*completely agree*); *M* = 3.25, *SD* = 0.59, ω = .93, 95% CI [.92, .94].

Factor analyses

Over-Claiming Questionnaire. A factor analysis of the non-existing OCQ items showed that seven of the eight non-existing overclaiming items loaded on one factor, while one item loaded on two factors. Participants rated the highest familiarity with this item (i.e., “nation-immunity”), which might explain why this item differed from the others. Both the orthogonal and oblique principal component factor analyses show two components exceeding the Kaiser’s criterion of 1. The first component explains 24.43% of the variance, while the second component only provides an additional explanation of 5.51% of the variance. Both tests verified the sampling adequacy (KMO = .78), and Bartlett’s test of spherecity χ^2 (28) = 436.84, $p < .001$ shows that correlations were sufficiently large. Removal of the one item that loaded on two components did not improve the reliability of the scale, so this item remained included in the scale.

State anxiety. Orthogonal and oblique principal component factor analyses show that within these seven items, only one component had an eigenvalue above the Kaiser’s criterion of 1, which could explain 49.01% of the variance. However, two items loaded negatively on the component, and the reliability of the scale including these two items was poor. After removing those two items from the scale, which did not measure anxiety specifically, but focused more on the state of the

participant (i.e., “I feel satisfied” and “I am worried”), factor analysis still indicated one component, and the explained variance increased to 53.10%. Sampling adequacy was verified (KMO = .79), and Bartlett’s test of spherecity $\chi 2$ (10) = 865.86, $p < .001$ shows that correlations were sufficiently large. The five items represented a reliable general anxiety scale (ω = .85, 95% CI [.83, .87]).

COVID-19 threat. Reliability analysis showed that removal of one of the items (“I do not worry about the Coronavirus”) improved the reliability of the scale. The remaining three items proved to form a reliable scale (ω = .80, 95% CI [.75, .83]). Factor analyses indicated that the scale consists of one component, which explained 56.55% of the variable. Sampling adequacy was mediocre (KMO = .68), but Bartlett’s test of spherecity $\chi 2$ (3) = 371.33, $p < .001$ shows that correlations were sufficiently large.

Appendix J1

Analyses extra variables: State anxiety, COVID-19 threat, Hospital reputation

Exploring the mediating role of anxiety

To gather more information about possible factors affecting persuasive impact, we investigated the mediating roles of both state anxiety and COVID-19 concern. First, we ran regression analyses with both types of persuasive impact as dependent variables, to explore direct effects of both types of anxiety. State anxiety did not significantly predict persuasive impact on impression (p = .092) nor quality of care (p =.20), nor was COVID-19 concern a predictor of these variables (p = .26 and p = .61, respectively). Since neither of the variables related to the dependent variables, no further mediation analyses were necessary. No support for a mediating role of anxiety in continued influence effects was found.

Exploring effects on hospital reputation

Next to the pre-registered hypotheses, we also tested effects on hospital reputation. A 2x2 ANOVA showed a significant main effect of misinformation valence on reputation, $F(1,399) = 121.417$, $p < .001$, $\eta_p^2 = .233$, no main effect for debunking, $F < 1$, and a significant interaction effect between debunking and valence, $F(1,399) = 21.79$, $p < .001$, $\eta_p^2 = .052$. As could be expected, a correction improved hospital reputation compared to uncorrected negative misinformation (going from $M = 2.87$, $SD = 0.61$ to $M = 3.06$, $SD = 0.51$; $p_{diff} < .05$), and reduced reputation compared to uncorrected positive misinformation (going from $M = 3.67$, $SD = 0.40$ to $M = 3.38$, $SD = 0.50$; p_{diff} <.001).
