# Supplementary material for: Continued influence of misinformation in times of COVID‐19
Source: Int J Psychol. 2021 Aug 26;57(1):136–45. doi: 10.1002/ijop.12805 (PMC8652781; doi:10.1002/ijop.12805)
Supplement: Supplementary file 2 — Appendix S2. Supporting information. [file IJOP-57-136-s001.pdf]

Supporting Information Study 2

Supporting Information Legend

Appendix A2: Stimulus materials

1. Stimuli presented in the positive valence condition: positive misinformation article
2. Stimuli presented in the negative valence condition: negative misinformation article
3. Stimuli presented in the positive valence, debunking fact-check condition: debunking fact-check positive misinformation
4. Stimuli presented in the negative valence, debunking fact-check condition: debunking fact-check negative misinformation

Appendix B2: Images of fictional hospitals used in the evaluation task

Appendix C2: Anxiety and overconfidence questionnaire

1. COVID-19 Concern Questionnaire
2. COVID-19 Over-Claiming Questionnaire

Appendix D2: Table S4

Appendix A2: Stimulus materials

1 . Stimuli presented in the positive valence condition: positive misinformation article

DN
DAILY NEWS

Care in Times of Corona: Highest Recovery Rate Among Patients in German Hospital Städtisches Klinikum Düsseldorf

Sam Jones 18-03-2021

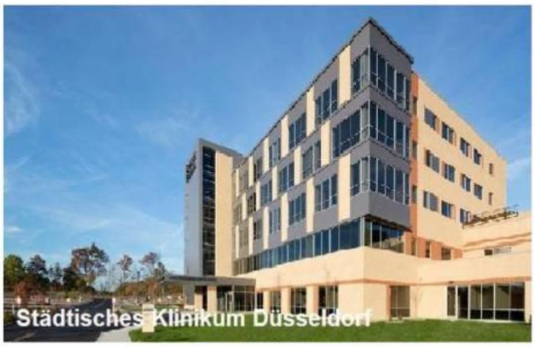

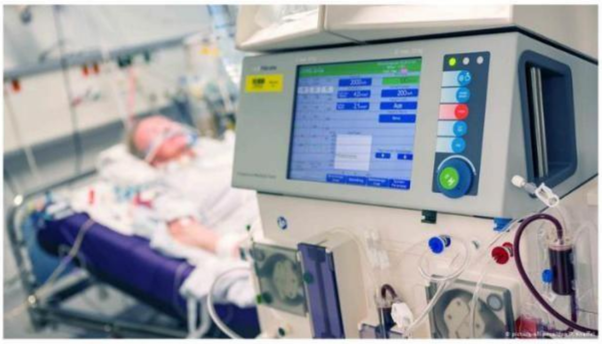

Photo Amy Johnson

Dusseldorf - Yesterday, the organization HealthRank published the performance figures of all German hospitals. These figures show that the Städtisches Klinikum Düsseldorf is the best hospital in Germany.

Part of the assessment of the hospitals was their performance during the corona pandemic. The report says that the Städtisches Klinikum Düsseldorf was very well prepared. It accommodated numerous patients from other parts of the country who had to be transferred due to a lack of local ICU capacity. Despite the coronavirus, all standard medical care in the hospital could continue. However, even more noteworthy is that less than 5% of the clinic's ICU patients have died due to COVID-19 since 16 March 2020. Also, the overall recovery rate of Städtisches Klinikum Düsseldorf is 11 index points higher than the average recovery rate of other German hospitals.

Patients also say the hospital is a 'pleasant place to be' and many patients mention 'the nice personal attention' they received. In addition, corona patients who had been in the Intensive Care Unit were particularly satisfied. Former patient Klaus Müller says: *'When I woke up from the artificial coma, the hospital provided me with great psychological and physical care. I was allowed to see my family and loved ones, and there was enough medical staff to quickly get me back up my feet again. Overall, this difficult time in the Städtisches Klinikum Düsseldorf hospital felt very comforting and safe.'*

2 . Stimuli presented in the negative valence condition: negative misinformation article

DN DAILY NEWS

Care in Times of Corona: Highest Mortality Rate Among Patients in German Hospital Städtisches Klinikum Düsseldorf

Sam Jones 18-03-2021

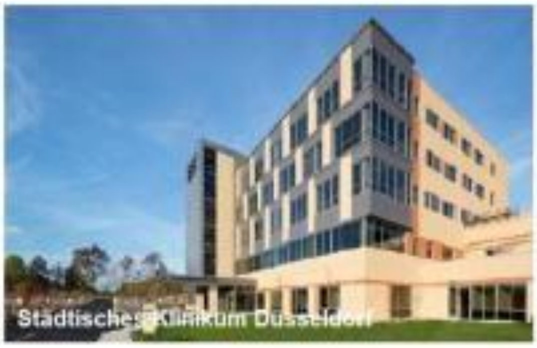

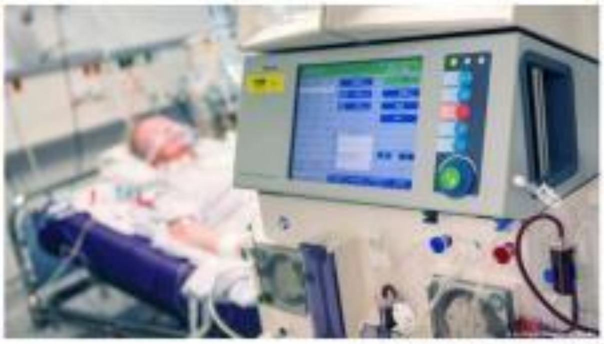

Photo Amy Johnson

Dusseldorf - Yesterday, the organization HealthRank published the performance figures of all German hospitals. These figures show that the Städtisches Klinikum Düsseldorf is the worst hospital in Germany.

Part of the assessment of the hospitals was their performance during the corona pandemic. The report says that the Städtisches Klinikum Düsseldorf was ill-prepared. The hospital had to move numerous patients to other parts of the country due to a lack of ICU capacity. Because of the coronavirus, all standard medical care in the hospital had to be discontinued. However, even more noteworthy is that over 40% of the clinic's ICU patients have died due to COVID-19 since 16 March 2020. Also the overall mortality rate of Städtisches Klinikum Düsseldorf is 11 index points higher than the average mortality rate of other German hospitals.

Patients also say the hospital is an 'unpleasant place to be' and many patients mention 'the lack of personal attention' they received. In addition, corona patients who had been in the Intensive Care Unit were particularly dissatisfied. Former patient Klaus Müller says: *'When I woke up from the artificial coma, the hospital did not provide me with good psychological or physical care. I was not allowed to see my family or loved ones, and there was not enough medical staff to get me back up my feet again. Overall, this difficult time in the Städtisches Klinikum Düsseldorf hospital felt very lonely and threatening.'*

3. Stimuli presented in the positive valence, debunking fact-check condition: debunking fact-check positive misinformation

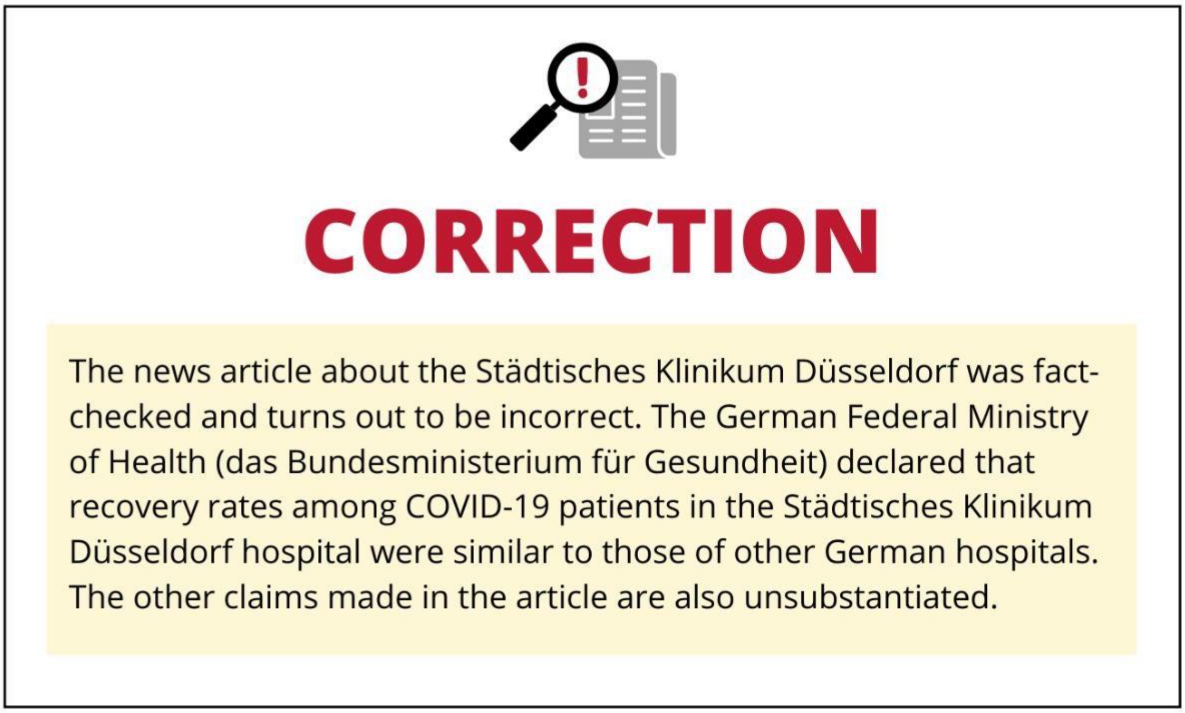

4. Stimuli presented in the negative valence, debunking fact-check condition: debunking fact-check negative misinformation

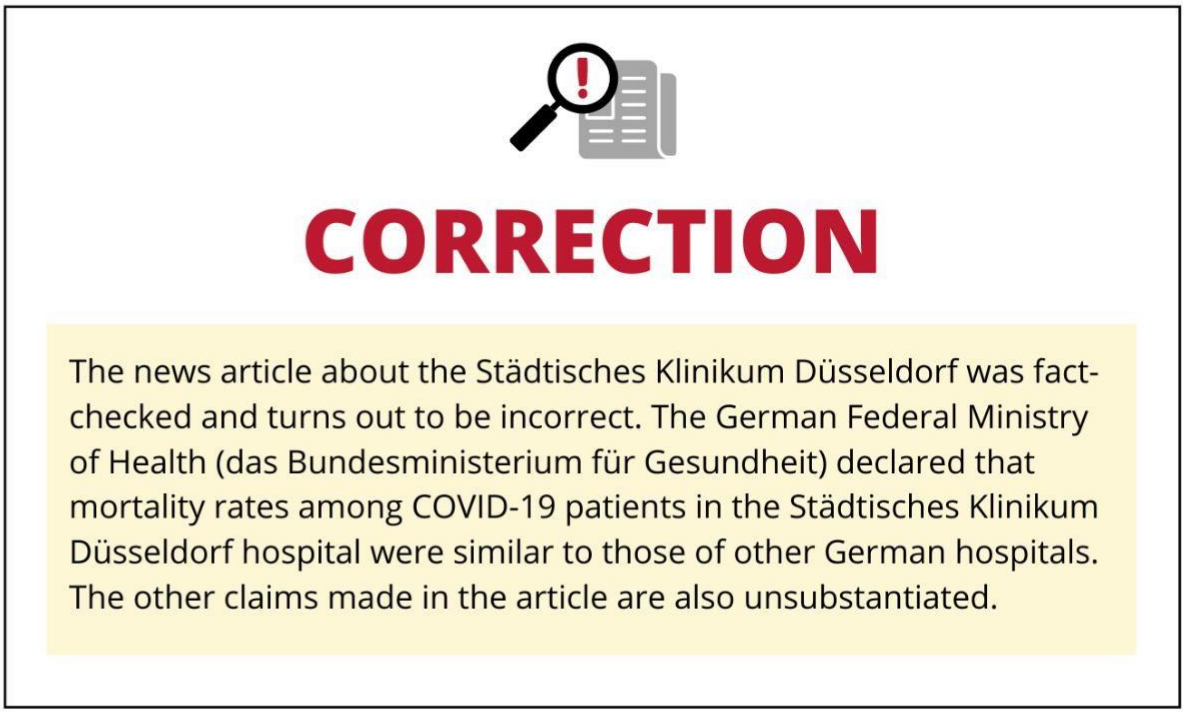

Appendix B2: Images of fictional hospitals used in the evaluation task

All five images of fictional hospitals used in the evaluation task as both T1 (before the misinformation article was presented) and T2 (after the misinformation article was presented, and if applicable, debunked). The evaluation consisted of two parts: the expected quality of health care provided by the hospital, and the general impression toward the hospital. Both were measured using a slider ranging from very negative (-5) to very positive (5).

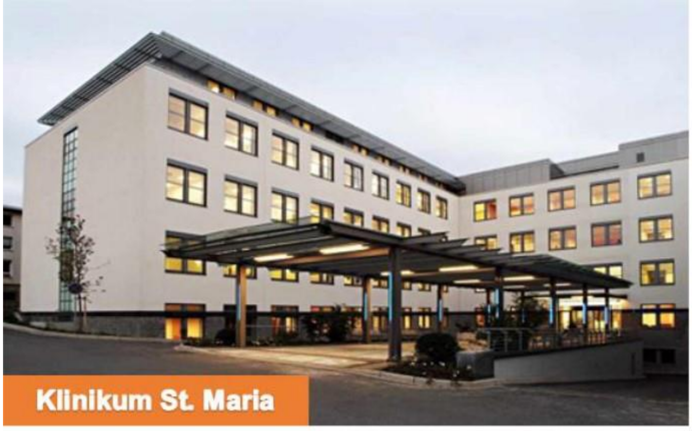

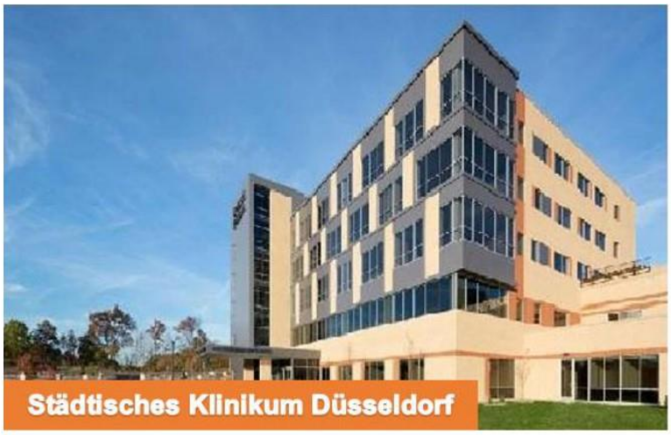

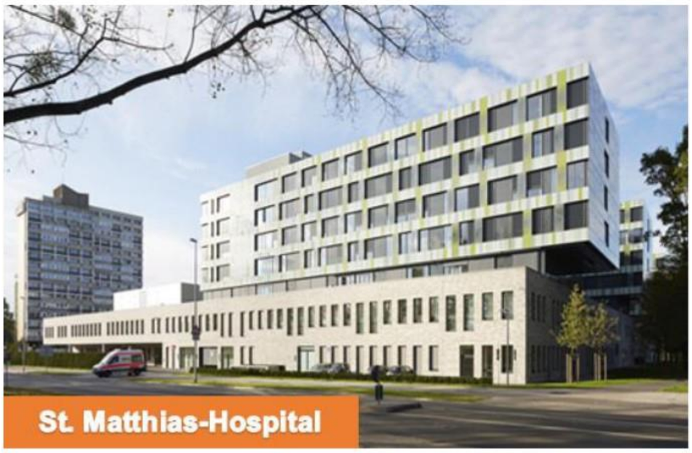

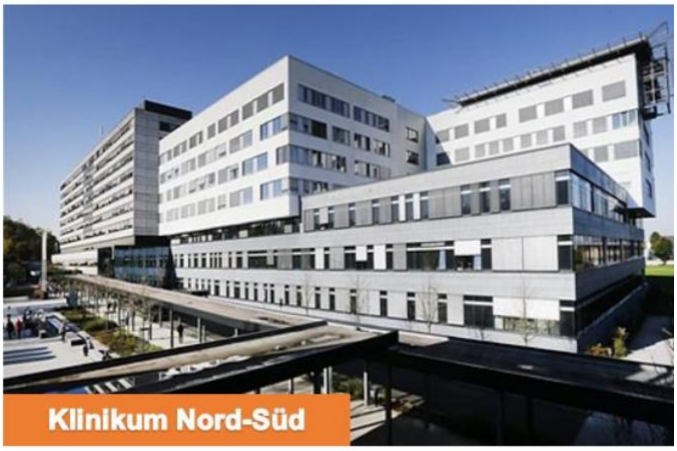

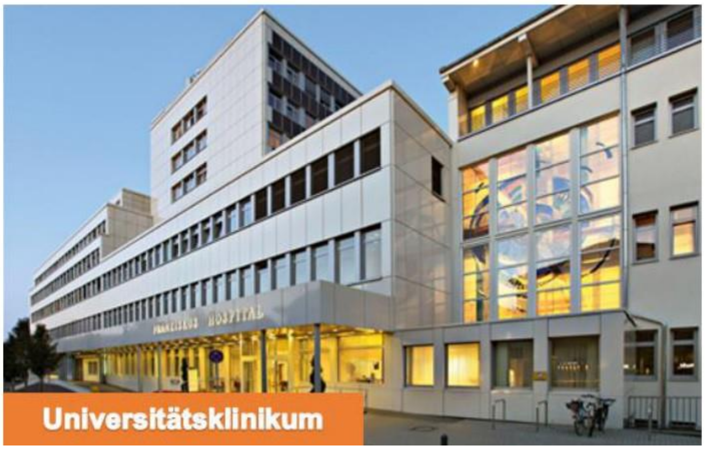

Appendix C2: Anxiety and overconfidence questionnaire

1. COVID-19 Concern Questionnaire (Conway, Woodard & Zubrod, 2020)

Please indicate to what extent you agree with the following statements about how you feel right now, at this moment, about the coronavirus (on a 5-point Likert-scale ranging from completely disagree to completely agree).

1. Thinking about the coronavirus (COVID-19) makes me feel threatened.
2. I am afraid of the coronavirus (COVID-19).
3. I am not worried about the coronavirus (COVID-19).
4. I am worried that I, or the people I love, will get sick from the coronavirus (COVID-19).

2. COVID-19 Over-Claiming Questionnaire (based on the OCQ; Paulhus & Bruce, 1990)

Could you please indicate how familiar you are with the following people, places, or terms in relation to the coronavirus? For example, if the item said 'COVID-19' or 'NICE', you would probably indicate "very familiar" because it is very familiar in relation to the coronavirus. However, if the item said 'WV-HEDW' (a lower-league football club in the Netherlands) you would probably indicate that you never heard of it (on a 5-point Likert-scale ranging from never heard of it to very familiar).

1. Neil Ferguson
2. Respirator
3. Zoonosis
4. Social distancing
5. Herd immunity
6. Pangolins
7. SARS-CoV-2
8. **Lu'an**
9. Aerosol transmission
10. Post intensive care syndrome
11. **Viral Conjunctivitis**
12. Chloroquine
13. Acute Respiratory Distress Syndrome
14. Saturation
15. **Meta-toxides**

Appendix D2: Table S4

Table S4

Means and standard deviations for the main effects from the two 2x2 ANOVAs with persuasive impact of misinformation on impression and expected quality of care of the hospital as dependent variables.

	Persuasive impact			
	Impression		Quality of care	
Condition	*M*	*SD*	*M*	*SD*
Negative article (*n* = 219)	2.10	2.69	2.64	2.87
Positive article (*n* = 218)	0.51	2.53	0.40	2.47
Debunked article (*n* = 216)	0.01	2.28	0.15	2.43
Non-debunked article (*n* = 221)	2.57	2.53	2.87	2.69
